# High-quality reduced graphene oxide-nanocrystalline platinum hybrid materials prepared by simultaneous co-reduction of graphene oxide and chloroplatinic acid

**DOI:** 10.1186/1556-276X-6-241

**Published:** 2011-03-21

**Authors:** Yinjie Wang, Jincheng Liu, Lei Liu, Darren Delai Sun

**Affiliations:** 1 School of Civil and Environmental Engineering, Nanyang Technological University, 639798, Singapore

## Abstract

Reduced graphene oxide-nanocrystalline platinum (RGO-Pt) hybrid materials were synthesized by simultaneous co-reduction of graphene oxide (GO) and chloroplatinic acid with sodium citrate in water at 80°C, of pH 7 and 10. The resultant RGO-Pt hybrid materials were characterized using transmission electron microscopy (TEM), powder X-ray diffraction (XRD), X-ray photoelectron spectroscopy (XPS), Fourier-transform infrared spectroscopy, and thermogravimetric analysis. Platinum (Pt) nanoparticles were anchored randomly onto the reduced GO (RGO) sheets with average mean diameters of 1.76 (pH 7) and 1.93 nm (pH 10). The significant Pt diffraction peaks and the decreased intensity of (002) peak in the XRD patterns of RGO-Pt hybrid materials confirmed that the Pt nanoparticles were anchored onto the RGO sheets and intercalated into the stacked RGO layers at these two pH values. The Pt loadings for the hybrid materials were determined as 36.83 (pH 7) and 49.18% (pH 10) by mass using XPS analysis. With the assistance of oleylamine, the resultant RGO-Pt hybrid materials were soluble in the nonpolar organic solvents, and the dispersion could remain stable for several months.

## Introduction

Graphene, a flat monolayer of two-dimensional honeycomb-structured carbon atoms, has attracted tremendous attention from both theoretical and experimental studies in recent years [1-6]. Its unique structural, mechanical, and electronic properties make it a promising candidate in many potential applications, such as sensors [7-9], electrodes [10-13], lithium storage [14,15], and hydrogen storage [16].

It is well known that carbon nanotubes (CNTs) deposited with platinum (Pt) nanoparticles exhibit outstanding catalytic activity [17-22]. Possessing similar properties, graphene has a larger surface area (theoretical value of ~2,600 m^2^/g [23] as unwrapped single-wall CNTs) than that of CNTs. Furthermore, graphene/reduced graphene oxide (RGO) can be produced at a lower cost through large-scale chemical synthesis [24,25]. All these advantages make the reduced graphene oxide-nanocrystalline platinum (RGO-Pt) hybrid materials even more attractive in engineering applications.

RGO-Pt hybrid materials have been successfully synthesized by several research groups [26-34]. One way to synthesize the RGO-Pt hybrid materials is to reduce the graphene oxide (GO) sheets deposited with Pt precursor by H_2 _[28,31]. Ethylene glycol [27,33,34] and sodium borohydride [29,30] have also been used as reducing agents to synthesize the RGO-Pt hybrid materials from the mixture of GO sheets and Pt precursors in the one-pot synthesis.

The monodispersity, the faceted uniformity, and the dispersion level of Pt nanocrystals significantly affect the high specificity in a catalytic process [35]. When used in the synthesis of the noble-metal nanocrystals [36-38], sodium citrate, a mild reducing agent and stabilizer, can control well the size and morphology of the nanocrystals.

**Figure 1:**
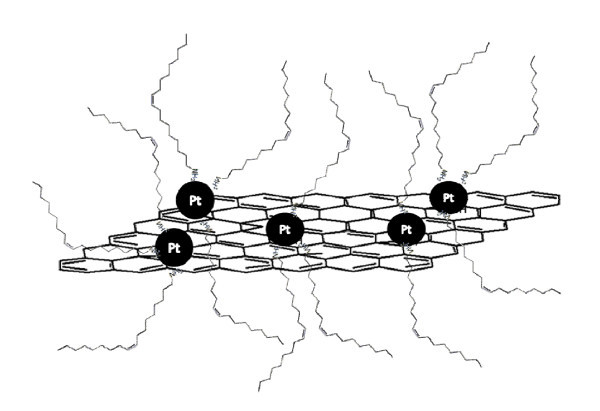
**A schematic illustration of the dispersed RGO-Pt hybrid materials assisted by OA molecules**.

In this article, the synthesis of the RGO-Pt hybrid materials is presented using sodium citrate as the reducing agent and the stabilizer at 80°C. To the best of our knowledge, this is the first report of sodium citrate being used for the reduction of GO. The resultant RGO-Pt hybrid materials are soluble in nonpolar organic solvents assisted by oleylamine (OA) (Figure 1), which will be extremely valuable for future catalytic applications. The detailed characterizations of the RGO-Pt hybrid materials carried out in this study provide us with an insight into the utilization of this new graphene-based material.

## Materials and methods

### Materials

Sodium nitrate (NaNO_3_, 99%), potassium permanganate (KMnO_4_, 99%), hydrogen peroxide (H_2_O_2_, 35%), concentrated sulfuric acid (H_2_SO_4_, 98%), oleylamine (C_18_H_36_NH_2_, 70%), sodium citrate dehydrate (Na_3_C_6_H_5_O_7_·2H_2_O, 99%), and concentrated hydrochloric acid (HCl, 36.5%) were purchased from Aldrich. Chloroplatinic acid (H_2_PtCl_6_·*x*H_2_O, with Pt content approximately 40%) was used as received from BDH Chemical. Natural graphite (SP-1) was purchased from Bay Carbon. All solvents were purchased from Merck. The deionized (DI) water was produced using the Millipore Milli-Q water purification system.

### Synthesis of RGO-Pt hybrid materials

The GO was synthesized according to the modified Hummers method [39], a process that had been described earlier [40]. The synthesis of the RGO-Pt hybrid material is a modification of the synthesis method of the Pt nanoparticles [41]. Fifty milligrams of as-prepared dried GO sample and 50 mg of H_2_PtCl_6_·*x*H_2_O were mixed in 50 mL of DI water under continuous stirring, purged with N_2 _at room temperature for 2 h, and the pH value of the mixture was approximately 2. The syntheses of two other batches, adjusted to the values of pH 7 and 10 by adding diluted NaOH solution, were carried out. Ten milliliters of 20 g/L sodium citrate solution was injected into the mixture after it was heated to 80°C. After being kept at 80°C for 24 h, the mixture was cooled to room temperature and filtered. The residue was washed with DI water and acetone before freeze-drying, and kept aside until further use. The resultant products were denoted as RGO-Pt-2, RGO-Pt-7, and RGO-Pt-10 for the samples prepared at pH 2, 7, and 10, respectively. Two control syntheses were carried out under the same experimental conditions as RGO-Pt-10 without adding either the Pt precursor or the GO; the dried samples were denoted as RGO-NoPt-10 and Pt-10, respectively.

Ten milligrams of the prepared RGO-Pt hybrid materials was dispersed in 10 mL of 1,2-dichlorobenzene (DCB) with the addition of OA (0.1-0.2 mL) under sonication for 30 min. The clear solution of RGO-Pt hybrid materials in DCB can remain stable for several months.

### Characterization

Transmission electron microscopy (TEM) and high-resolution TEM (HRTEM) images were obtained using a JEOL JEM-2010 operated at 200 kV. The samples for the analysis were prepared by dropwise addition of dilute DCB solutions of the RGO-Pt hybrid materials onto carbon-coated copper grids. Powder X-ray diffraction (XRD) patterns were obtained using Bruker AXS D8 Advance (Cu Kα λ = 1.5406 Å). X-ray photoelectron spectroscopy (XPS) analysis was performed in an ultrahigh vacuum chamber, with a base pressure below 2.66 × 10^-7 ^Pa at room temperature. Photoemission spectra were recorded using a Kratos Axis Ultra spectrometer equipped with a standard monochromatic Al Kα excitation source (*h*ν = 1486.71 eV). Fourier-transform infrared spectroscopy (FTIR) analysis was performed using a Perkin Elmer GX with potassium bromide dye for the preparation of the sample pellet. Thermogravimetric analysis (TGA) was carried out under air using thermogravimetric analyzer Perkin Elmer TGA 7. The samples were heated from room temperature to 900°C at a ramp rate of 10°C/min.

## Results and discussion

The RGO-Pt hybrid materials were prepared from the co-reduction of GO and H_2_PtCl_6 _at 80°C at different pH values. The as-prepared RGO-Pt-2 appears brownish yellow and slightly darker than the pristine GO, while RGO-Pt-7 and RGO-Pt-10 show black color. This difference in color could be attributed to the reduction degree of the original GO sheets and the formation of the Pt nanoparticles. The RGO-Pt-2 can be easily dispersed in DI water, while RGO-Pt-7 and RGO-Pt-10 can be stably dispersed in nonpolar organic solvents assisted by OA.

**Figure 2:**
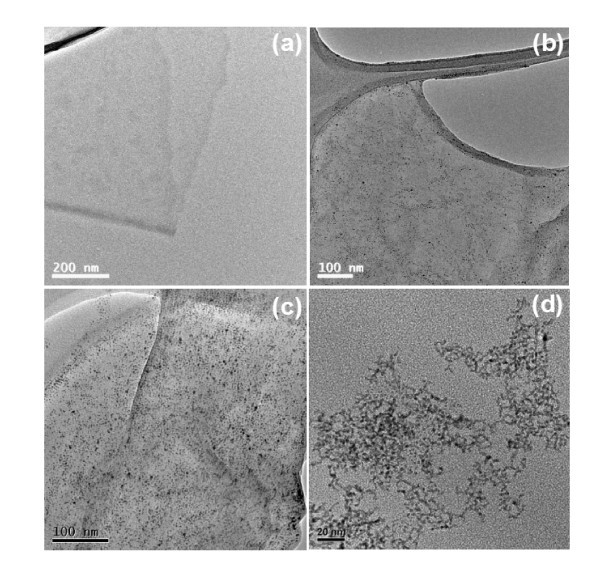
**TEM images of (a) RGO-Pt-2, (b) RGO-Pt-7, (c) RGO-Pt-10, and (d) Pt-10**.

Figure 2 shows the TEM images of the RGO-Pt hybrid materials prepared at different pH values of 2, 7, and 10 and the Pt nanoparticles synthesized at pH 10. At pH 2 (Figure 2a), no Pt nanoparticles are observed on the GO sheets. This could be because citrate is unable to reduce H_2_PtCl_6 _in this acidic condition [42]. At pH 7 (Figure 2b) and pH 10 (Figure 2c), the RGO sheets are randomly decorated with Pt nanoparticles with diameters of the order of a few nanometers. The density of the Pt nanoparticles for RGO-Pt-10 is higher than that forRGOPt-7. Few aggregates of the Pt nanoparticles are observed, suggesting the strong interactions between the Pt nanoparticles and the RGO sheets [27,43]. However, without the support of the RGO sheets, the Pt nanoparticles synthesized at pH 10 are aggregated together, as shown in Figure 2d. This suggests the important role RGO plays in the co-reduction process to obtain unaggregated Pt nanoparticles.

**Figure 3:**
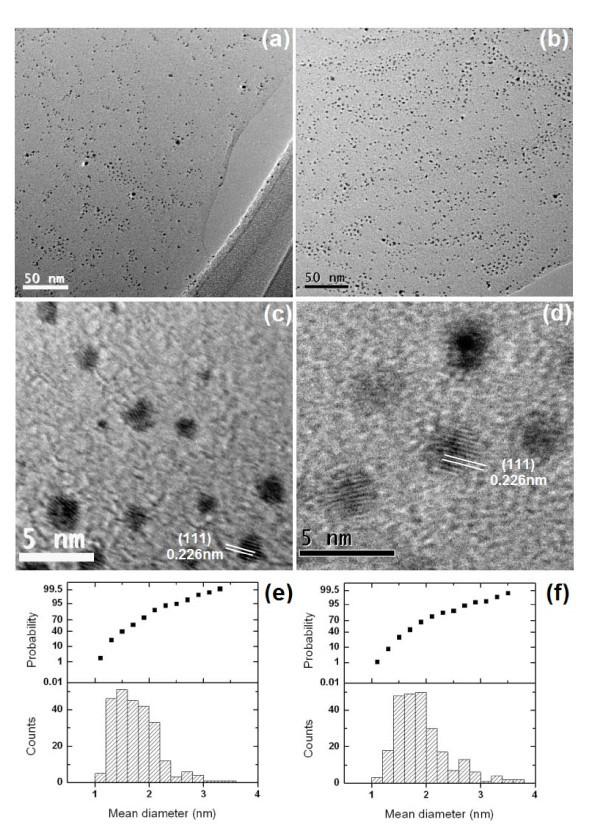
**TEM images of (a) RGO-Pt-7 and (b) RGO-Pt-10; HRTEM images of (c) RGO-Pt-7 and (d) RGO-Pt-10; and Pt particle-size counts and probability curves of (e) RGO-Pt-7 and (f) RGO-Pt-10 (250 particles were randomly selected for the calculation from (a) and (b), respectively)**.

Enlarged TEM images (Figure 3a, b) show that the Pt nanoparticles on the graphene sheets are mostly quasi-spherical in shape. Figure 3e, f, shows the size distribution analysis performed by selecting 250 Pt nanoparticles from Figure 3a, b, respectively. For both the samples prepared at pH 7 and 10, the mean diameters of the Pt nanoparticles fall within the range of 1-4 nm, and it is mainly between 1 and 2 nm. Furthermore, no significant difference in the sizes of the Pt nanoparticles was observed for the samples synthesized at these two pH values.

The average mean diameters of the Pt nanoparticles are 1.76 and 1.93 nm for RGO-Pt-7 and RGO-Pt-10, respectively. The high-resolution TEM (HRTEM) images are shown in Figure 3c, d. The lattice spacing in both samples (RGO-Pt-7 and RGO-Pt-10) is measured as 0.226 nm, which corresponds to the (111) planes of the face-centered cubic Pt [34].

**Figure 4:**
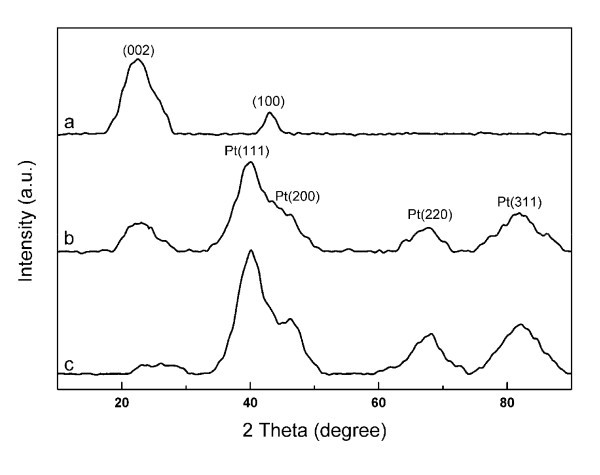
**XRD patterns of RGO-Pt hybrid materials**: **(a) **RGO-NoPt-10, **(b) **RGO-Pt-7, and **(c) **RGO-Pt-10.

**Figure 5:**
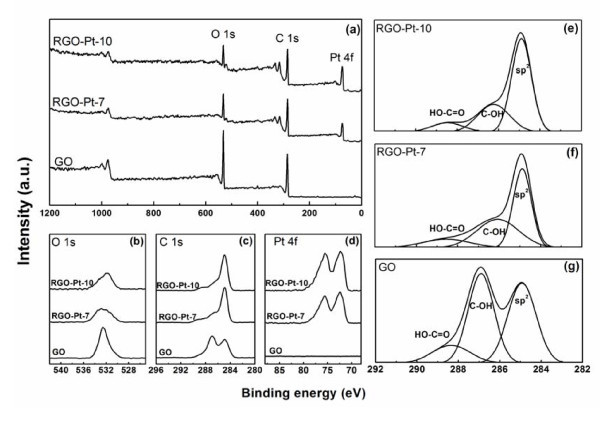
**XPS spectra of GO, RGO-Pt-7, and RGO-Pt-10**: **(a) **survey scan, high-resolution scan in the **(b) **O *1 s *region, **(c) **C *1 s *region, and **(d) **Pt *4f *region; C *1 s *XPS spectra of: **(e) **RGO-Pt-10, **(f) **RGO-Pt-7, and **(g) **GO indicate oxidation with three major components that correspond to carbon atoms in different functional groups: the *sp*^2^-hybridized C-C, the C in C-OH bonds, and the carboxylate carbon.

Figure 4 shows the XRD patterns of the RGO-NoPt-10, RGO-Pt-7, and RGO-Pt-10 powders. In Figure 4a, the characteristic peak at 2θ = 22.6° is ascribed to the (002) planes of the chemically reduced GO stacks [33], indicating the effective reduction of GO by citrate sodium at pH 10. The (002) diffraction intensity of RGO-Pt-7 at 2θ = 22.6° is lower than that of RGO-NoPt-10. Moreover, no obvious (002) diffraction peak is observed in the XRD pattern of RGO-Pt-10 because the regular stacks of RGO are destroyed by the intercalation of Pt nanoparticles [34]. In the XRD patterns of RGO-Pt-7 and RGO-Pt-10, the clear diffraction bands centered at 2θ of 39.9°, 46.3°, 67.7°, and 81.4° are corresponding to the (111), (200), (220), and (311) reflections of Pt nanocrystals [26], respectively. The broadening peaks are caused by the small size of the Pt nanoparticles. The size of the Pt nanoparticles can be estimated by means of the Scherrer equation based on the full-width at half-maximum of Pt (111) peak at 2θ = 39.7°. The calculated average sizes of the Pt nanoparticles are 1.97 and 2.07 nm for RGO-Pt-7 and RGO-Pt-10, respectively, which are in good agreement with TEM observations of this study. Elemental analysis based on the XPS data (Figure 5a) reveals that the O/C atomic ratios are 0.3738, 0.1889, and 0.1816 for GO, RGO-Pt-7, and RGO-Pt-10, respectively. The Pt mass loadings are 36.83 and 49.18% for RGO-Pt-7 and RGO-Pt-10. The reduced O/C atomic ratio and the decrease in the intensity of the O *1 s *scan (Figure 5b) indicate the effective reduction of the GO sheets at pH 7 and 10. High-resolution spectra of the C *1 s *region in Figure 5c, e-g show that both epoxide and hydroxyl functional groups are considerably decreased for RGO-Pt-7 and RGO-Pt-10. The Pt *4f *7/2 peak and Pt *4f *5/2 peak have been shifted to higher energy levels of 72.4 and 75.6 eV, respectively, for both the samples, which may be due to the interaction between the Pt nanoparticles and the reduced graphene sheets. The existence of Pt *4f *signals (Figure 5a, d) observed for RGO-Pt-7 and RGO-Pt-10 further confirms that the RGO-Pt hybrid materials have been synthesized successfully [44].

**Figure 6:**
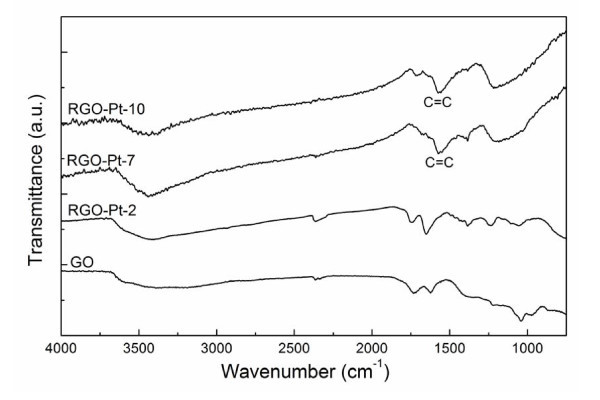
**FTIR spectra of the GO, RGO-Pt-2, RGO-Pt-7, and RGO-Pt-10**.

Figure 6 presents the FTIR spectra of GO, RGO-Pt-2, RGO-Pt-7, and RGO-Pt-10. For the spectrum of GO, the broad band ranging from 3600 to 3000 cm^-1 ^corresponds to the O-H stretching vibrations of adsorbed water molecules [45]. The bands at 1730 and 1039 cm^-1 ^are assigned to the C = O and C-OH stretching vibrations of COOH groups, respectively [46]. The band at 1620 cm^-1 ^is assigned to the vibration of the adsorbed water molecules as well as to the contributions from the skeletal vibrations of un-oxidized graphitic domains [47]. The spectrum of RGO-Pt-2 is similar to that of GO, indicating that the GO sheets were not reduced at pH 2. For the spectra of RGO-Pt-7 and RGO-Pt-10, the broad bands centered at 1212 and 1218 cm^-1^, respectively, are ascribed to the C-O stretching vibrations of the phenolic and carboxylic groups from the reduced GO sheets [48,49]. Moreover, the broad bands at 1574 cm^-1 ^for both the samples are assigned to the C = C skeletal vibration of graphene sheets [45], which confirms the successful reduction of GO sheets.

**Figure 7:**
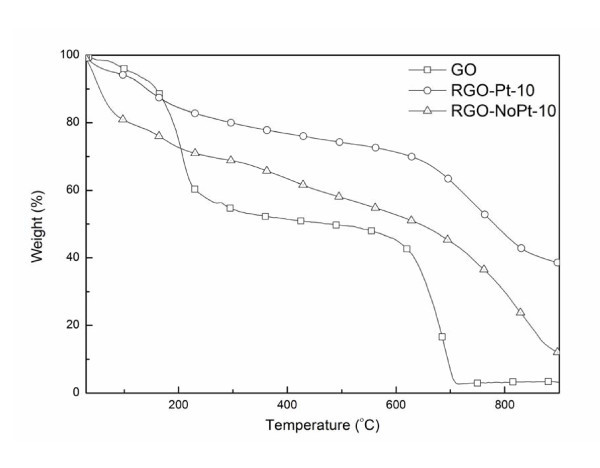
**TGA curves of the GO, RGO-Pt-10, and RGO-NoPt-10**.

In Figure 7, the TGA curve of GO shows significant mass losses at 200 and 650°C due to the removal of oxygen-containing groups and carbon oxidation [40], respectively. These two major losses appear much smaller for RGO-Pt-10 and RGO-NoPt-10, which indicates the successful deoxygenation of GO at pH 10. The nearly parallel curves of RGO-Pt-10 and RGO-NoPt-10 suggest that the attachment of the Pt nanoparticles onto the graphene sheet did not lessen the thermal stability of the graphene sheets.

The characterizations confirm that the RGO-Pt hybrid materials can be produced by a simultaneous co-reduction process at pH 7 and 10. In the acidic (pH 2) condition, neither GO nor the Pt precursor could be reduced by the reductant sodium citrate; however, in neutral (pH 7) and basic (pH 10) conditions, sodium citrate was able to reduce the GO to graphene and the Pt precursor to Pt nanoparticles. This could be explained by the release of H^+ ^and CO_2 _during the reduction process involving sodium citrate as the reducing agent [50]. The increase of the pH values could consume the H^+ ^and hence favors the reduction reaction. However, in the lower pH region, the high concentration of H^+ ^would inhibit the reduction reaction from taking place.

The as-prepared RGO-Pt hybrid materials could not be dispersed well in any polar and nonpolar solutions because the oxygen-containing groups were decreased in the reduction process. However, the RGO-Pt hybrid materials assisted by OA could be dispersed well in nonpolar solvents such as DCB, toluene, and chlorobenzene. The dispersion can remain stable for several months. The dispersion of the RGO-Pt hybrid materials in DCB is caused by the coordination between OA and Pt nanoparticles, which is similar to the OA-assisted dispersion of CNT [51-53]. The weak nucleophilicity of OA ensures that the Pt nanoparticles are kept bonded together with the reduced GO. In order to investigate the interaction between OA and reduced GO, the dispersion of the RGO-NoPt-10 was attempted in DCB with the assistance of OA. It is worth mentioning that the RGO-NoPt-10 could not be dispersed satisfactorily in DCB even with the assistance of OA. Therefore, it is believed that the intercalation of Pt nanoparticles into the stacked graphene sheets and the coordination with OA are crucial for the redispersion of RGO-Pt hybrid materials. As a weak ligand, OA can also be very easily washed away by common solvents, like ethanol [54,55], hexane [56], and acetic acid [55].

## Conclusions

It has been shown that RGO-Pt hybrid materials can be synthesized by simultaneous co-reduction of GO and H_2_PtCl_6 _with sodium citrate at modest temperatures in neutral and basic conditions. The attachment of Pt nanoparticles onto reduced GO sheets can effectively prevent the aggregation of GO during reduction. The reduced GO can work as excellent supporters for dispersing and stabilizing the Pt nanoparticles. The resultant RGO-Pt hybrid materials exhibit high thermal stability, and are soluble in nonpolar organic solvents with the assistance of OA. The dispersion of RGO-Pt hybrid material composite is important for future catalytic applications. This material could be useful for the fabrication of the thin film that can replace the Pt coating in the counter electrode of the dye-sensitized solar cells. It could also be used as the electrode material for proton exchange membrane fuel cells.

## Abbreviations

CNTs: carbon nanotubes; DI: deionized; DCB: 1,2-dichlorobenzene; FTIR: Fourier-transform infrared spectroscopy; GO: graphene oxide; HRTEM: high-resolution TEM; OA: oleylamine; Pt: Platinum; RGO: reduced GO; RGO-Pt: reduced graphene oxide-nanocrystalline platinum; TEM: transmission electron microscopy; X-ray diffraction (XRD): X-ray photoelectron spectroscopy (XPS).

## Competing interests

The authors declare that they have no competing interests.

## Authors' contributions

YW and JL did the synthetic and characteristic job in this journal. JL gave the advice and guide the experiment including the using of oleylamine to disperse the RGO-Pt composite in organic solvents. LL helped in the test of FTIR. For the writing of manuscript, YW wrote the manuscript, revised by JL and prof DS. LL did some spelling check.
